# The Beta Adrenergic Receptor Blocker Propranolol Counteracts Retinal Dysfunction in a Mouse Model of Oxygen Induced Retinopathy: Restoring the Balance between Apoptosis and Autophagy

**DOI:** 10.3389/fncel.2017.00395

**Published:** 2017-12-12

**Authors:** Maurizio Cammalleri, Filippo Locri, Elisabetta Catalani, Luca Filippi, Davide Cervia, Massimo Dal Monte, Paola Bagnoli

**Affiliations:** ^1^Department of Biology, University of Pisa, Pisa, Italy; ^2^Department for Innovation in Biological, Agro-Food and Forest Systems, University of Tuscia, Viterbo, Italy; ^3^Neonatal Intensive Care Unit, Medical Surgical Fetal-Neonatal Department, Meyer University Children’s Hospital, Florence, Italy

**Keywords:** beta adrenergic receptors, propranolol, proliferative retinopathies, retinal neurons, apoptosis, autophagy, electroretinogram

## Abstract

In a mouse model of oxygen induced retinopathy (OIR), beta adrenergic receptor (BAR) blockade has been shown to recover hypoxia-associated retinal damages. Although the adrenergic signaling is an important regulator of apoptotic and autophagic processes, the role of BARs in retinal cell death remains to be elucidated. The present study was aimed at investigating whether ameliorative effects of BAR blockers may occur through their coordinated action on apoptosis and autophagy. To this aim, retinas from control and OIR mice untreated or treated with propranolol, a non-selective BAR1/2 blocker, were characterized in terms of expression and localization of apoptosis and autophagy markers. The effects of propranolol on autophagy signaling were also evaluated and specific autophagy modulators were used to get functional information on the autophagic effects of BAR antagonism. Finally, propranolol effects on neurodegenerative processes were associated to an electrophysiological investigation of retinal function by recording electroretinogram (ERG). We found that retinas of OIR mice are characterized by increased apoptosis and decreased autophagy, while propranolol reduces apoptosis and stimulates autophagy. In particular, propranolol triggers autophagosome formation in bipolar, amacrine and ganglion cells that are committed to die by apoptosis in response to hypoxia. Also our data argue that propranolol, through the inhibition of the Akt-mammalian target of rapamycin pathway, activates autophagy which decreases retinal cell death. At the functional level, propranolol recovers dysfunctional ERG by recovering the amplitude of a- and b-waves, and oscillatory potentials, thus indicating an efficient restoring of retinal transduction. Overall, our results demonstrate that BAR1/2 are key regulators of retinal apoptosis/autophagy, and that BAR1/2 blockade leads to autophagy-mediated neuroprotection. Reinstating the balance between apoptotic and autophagic machines may therefore be viewed as a future goal in the treatment of retinopathies.

## Introduction

In several retinal disorders, apoptotic pathways are primarily involved in retinal cell death leading to progressive visual dysfunction (Chinskey et al., 2014). In addition, recent data also demonstrate an important role of autophagy in eye diseases thus representing a new emerging area of research (Boya et al., 2016). Autophagy is a catabolic pathway that promotes the degradation and recycling of cellular components. It functions in retinal cell death, but also serves as a cell survival mechanism and its modulation may be either beneficial or deleterious depending on the retinal cell type involved and the disease context (Mitter et al., 2012; Russo et al., 2013; Chinskey et al., 2014; Frost et al., 2014; Boya et al., 2016; Chai et al., 2016; Rosa et al., 2016; Amato et al., 2017). In this respect, drugs targeting the autophagic pathway may provide a new therapeutic strategy to ameliorate retinal disorders.

Adrenergic signaling is an important regulator of apoptotic and autophagic processes (Branco et al., 2014; Wauson et al., 2014), but its role in retinal disorders is poorly understood. There is a number of studies demonstrating that beta adrenergic receptor (BAR) blockers may function to counteract several eye disorders as demonstrated in rodent models of diabetic retinopathy (DR) (Nassiri et al., 2016), age-related macular degeneration (AMD) (Lavine et al., 2013; Nourinia et al., 2015; Nassiri et al., 2016) and oxygen induced retinopathy (OIR) (Martini et al., 2011; Ristori et al., 2011; Dal Monte et al., 2013b; Casini et al., 2014). In the OIR model that very closely recapitulates the pathologic events occurring in retinopathy of prematurity (ROP) (Stahl et al., 2010), mice are exposed to hyperoxia from postnatal day (PD)7 to PD12 when, returning to normoxia, they undergo to relative hypoxia that is felt as ischemia (Smith et al., 1994). In this model, BAR blockade with propranolol, a BAR1/2 non-selective blocker, counteracts retinal neovessel growth in response to hypoxia (Ristori et al., 2011; Dal Monte et al., 2013b) indicating that BAR activation may promote angiogenesis. These studies have contributed to lay the ground for several independent clinical trials that have demonstrated the effectiveness of propranolol in counteracting the disease progression in preterm newborns suffering from ROP (Filippi et al., 2013, 2017; Makhoul et al., 2013; Bancalari et al., 2016).

The OIR mice model is also characterized by profound visual dysfunction as determined by altered electroretinogram (ERG) (Fulton et al., 2009) that would result from massive retinal cell loss that is initiated by apoptotic events at PD12 and culminates around PD14/15 (Sennlaub et al., 2002; Vecino et al., 2004; Narayanan et al., 2011).

Currently, little is known about the potential role of autophagy in retinal cell death that characterizes OIR mice. Whether modulation of retinal responses to hypoxia involves a coordinated action of BARs on apoptosis and autophagy remains to be established. In the present study, we addressed this issue by investigating whether BAR blockade with propranolol ameliorates OIR-associated retinal damage by preventing retinal cell degeneration through a coordinated action on apoptosis and autophagy. To this aim retinas of untreated or propranolol-treated mice, also in the presence of specific autophagy modulators, were characterized in terms of apoptosis and autophagy at different times after hyperoxia. Propranolol effects on neurodegenerative processes were associated to an electrophysiological investigation of retinal function by recording ERG.

## Materials and Methods

### Reagents

The cocktails of protease and phosphatase inhibitors (Complete and PhosSTOP) were from Roche Diagnostics (Milan, Italy). The primary antibodies, including their suppliers, are listed in **Table 1**. The horseradish peroxidase secondary antibodies were obtained from Cell Signaling Technology (Beverly, MA, United States). All other chemicals including propranolol [(±)-1-Isopropylamino-3-(1-naphthyloxy)-2-propanol hydroch loride], rapamycin (23,27-Epoxy-3H-pyrido[2,1-c][1,4]oxaazacyclohentriacontine) and wortmannin [(1alpha,11alpha)-11-(Acetyloxy)-1-(methoxymethyl)-2-oxaandrosta-5,8-dieno(6,5,4-bc)furan-3,7,17-trione] were from Sigma–Aldrich (St. Louis, MO, United States).

**Table 1:** List of primary antibodies.

Antibody	Dilution	Source	Catalog
Mouse monoclonal anti-cytochrome c	1:500 (WB)	BD Biosciences	556433
Rabbit monoclonal anti-cleaved Caspase 3	1:1,000 (WB)	Cell Signaling Technology	9664
Rabbit polyclonal anti-cleaved Caspase 3	1:400 (IF)	Sigma–Aldrich	C8487
Rabbit polyclonal anti-p62/SQSTM1	1:200 (WB)	Sigma–Aldrich	P0068
Rabbit polyclonal anti-p62/SQSTM1	1:200 (IF)	Sigma–Aldrich	P0067
Rabbit polyclonal anti-LC3A/B	1:500 (WB)	Cell Signaling Technology	4108
Rabbit polyclonal anti-LC3	1:100 (IF)	Sigma–Aldrich	L8918
Rabbit polyclonal anti-Akt	1:1,000 (WB)	Cell Signaling Technology	9272
Rabbit polyclonal anti-p-S6 (Ser240/244)	1:1,000 (WB)	Cell Signaling Technology	2215
Mouse monoclonal anti-S6	1:1,000 (WB)	Cell Signaling Technology	2317
Rabbit monoclonal anti-p-4EBP1 (Thr37/46)	1:1,000 (WB)	Cell Signaling Technology	2855
Rabbit monoclonal anti-4EBP1	1:1,000 (WB)	Cell Signaling Technology	9644
Rabbit polyclonal anti-p-Ulk1 (Ser757)	1:1,000 (WB)	Cell Signaling Technology	6888
Rabbit monoclonal anti-p-Ulk1 (Ser555)	1:1,000 (WB)	Cell Signaling Technology	5869
Rabbit monoclonal anti-Ulk1	1:1,000 (WB)	Cell Signaling Technology	8054
Rabbit monoclonal anti-p-AMPKα (Thr172)	1:1,000 (WB)	Cell Signaling Technology	2535
Rabbit monoclonal anti-AMPKα	1:1,000 (WB)	Cell Signaling Technology	5832
Rabbit monoclonal anti-β-actin	1:2,500 (WB)	Sigma–Aldrich	A2228
Mouse monoclonal anti-PKC	1:200 (IF)	Sigma–Aldrich	P5704
Mouse monoclonal MAb 115A10	1:200 (IF)	Shinobu C. Fujita, (Japan)	
Rabbit polyclonal anti-GAT-1	1:400 (IF)	Merck Millipore	AB1570
Rabbit polyclonal anti-Dab1	1:300 (IF)	Sigma–Aldrich	SAB4503448
Mouse monoclonal anti-β-Tubulin III	1:400 (IF)	Sigma–Aldrich	T8660
AffiniPure Fab fragment goat anti-rabbit IgG (H+L)	1:50 (IF)	Jackson ImmunoResearch	JI111007003
Fluorescein (FITC)-AffiniPure Fab fragment goat anti-rabbit IgG (H+L)	1:400 (IF)	Jackson ImmunoResearch	JI111097003


*WB, western blot; IF, immunofluorescence.*

### Animals

Two month-old male and female mice (C57BL/6J strain) were originally purchased from Charles River Laboratories Italy (Calco, Italy) and were mated in our breeding colony. Mice were housed in a regulated environment (23 ± 1°C, 50 ± 5% humidity) with a 12 h light/dark cycle (lights on at 08.00 a.m.), and provided with food and water *ad libitum*. This study was carried out in strict accordance with the recommendations in the Guide for the Care and Use of Laboratory Animals of the National Institutes of Health and adheres to the ARVO Statement for the Use of Animals in Ophthalmic and Vision Research. Procedures were carried in compliance with the Italian guidelines for animal care (DL 116/92) and the European Communities Council Directive (86/609/EEC), and were approved by the Committee on the Ethics of Animal Experiments of the University of Pisa (Permit Number: 0009069/2014). All efforts were made to reduce both animal suffering and the number of animals used.

### Mouse Model of OIR and Pharmacological Treatment

In a typical model of OIR, litters of mouse pups (*n* = 162) with their nursing mothers were exposed in an infant incubator to high oxygen concentration (75% ± 2%) between PD7 and PD12 before returning to room air between PD12 and PD17 (Smith et al., 1994). The data were collected from both males and females and the results combined, as there was no apparent gender difference.

In the present study, propranolol at 20 mg/kg/dose was dissolved in citrate buffer (vehicle) and was given subcutaneously three times a day from PD12 to PD16. Propranolol was also administered only at PD16 (acute treatment). Sham injections were performed with vehicle. In all experiments, no differences were observed between untreated and vehicle-treated retinas. In previous studies using the OIR mice model (Ristori et al., 2011), we demonstrated that the pathological signs of OIR were dose-dependently ameliorated by subcutaneous propranolol with no effects at 0.2 mg/kg/dose, moderate effects at 2 mg/kg/dose and maximal effects at 20 mg/kg/dose. This dose results in a concentration of about 18 ng/mg retina as demonstrated by liquid chromatography-mass spectrometry (Dal Monte et al., 2013b). Also, the subcutaneous injections of the BAR2 selective blocker ICI-118,551 have been shown to be effective in counteracting pathological signs of OIR (Martini et al., 2011). Of notice, systemic propranolol at 20 mg/kg/dose is apparently safe since it acts on the retina without any effect in the brain or those organs, such as lungs and heart, known to be targeted by BAR blockers (Ristori et al., 2011).

Intravitreal injection were performed at PD12 in OIR mice anesthetized by intraperitoneal injection of Avertin (1.2% tribromoethanol and 2.4% amylene hydrate in distilled water, 0.02 ml/g body weight). In particular, rapamycin [4 mM in 1 μl phosphate buffer saline (PBS) containing 2.5% dimethyl sulfoxide (DMSO)] and wortmannin (0.5 mM in 1 μl PBS containing 2.5% DMSO) (Aoki et al., 2015; Liu et al., 2016) were administered intravitreally using a microsyringe (NanoFil syringe; World Precision Instruments, Sarasota, FL, United States). Pupils were dilated using topical 0.5% atropine. Rapamycin or wortmannin were injected into the left eye, while the right eye was injected with PBS and served as a control.

Anesthetized mice were sacrificed at PD13 (*n* = 70), PD14 (*n* = 20), PD15 (*n* = 20), PD16 (*n* = 20), or PD17 (*n* = 32). For each experiments and data analysis, at least four different littermates were used.

### Western Blot Analysis

Protein expression was analyzed following published protocols (Cervia et al., 2002, 2003, 2007, 2016; Cazzato et al., 2014; De Palma et al., 2014; Lulli et al., 2015; Amato et al., 2017). Briefly, pooled sample retinas (2 retinas from 2 mice for each experimental condition) were sonicated in 10 mM Tris-HCl (pH 7.6) containing 5 mM EDTA, 3 mM EGTA, 250 mM sucrose, 10% SDS, and supplemented with a cocktail of protease and phosphatase inhibitors. Homogenates were then centrifuged at 22,000 *g* for 15 min at 4°C. The supernatants, containing cytosolic proteins, were used. Equal amounts of proteins were separated by 4–20% SDS-polyacrylamide gel electrophoresis gels (Criterion TGX Stain-free precast gels; Bio-Rad Laboratories, Hercules, CA, United States) and transferred onto nitrocellulose membrane using a Bio-Rad Trans-Blot Turbo System. The membranes were then probed using the primary antibodies listed in **Table 1**. After the incubation with the appropriate horseradish-peroxidase-conjugated secondary antibody, bands were visualized using the Clarity Western ECL substrate with a ChemiDoc XP imaging system (Bio-Rad Laboratories). Bands were quantified for densitometry using the Image Lab software (Bio-Rad Laboratories) and normalized to β-actin. When appropriate, primary antibodies that recognize the protein independently of its phosphorylation state, i.e., S6 ribosomal protein (S6), eukaryotic initiation factor 4E-binding protein-1 (4EBP1), Unc-51 Like Autophagy Activating Kinase 1 (Ulk1) and AMP-activated protein kinase (AMPK), were also used in reprobing experiment for normalization purposes.

### Immunofluorescence and Confocal Microscopy

Eye-cups were immersion-fixed for 1.5 h in 4% paraformaldehyde in 0.1 M PBS at 4°C, transferred to 25% sucrose in 0.1 M PBS, and stored at 4°C. Retinal sections (10 μm thick) were cut on a cryostat, mounted onto positive charged slides and stored at -20°C until use. For immunostaining (Catalani et al., 2009; Amato et al., 2017), sections were washed in PBS and then pre-incubated for 15 min at room temperature with 5% bovine serum albumin (BSA; Life Technologies, Monza, Italy) and 10% of normal goat serum (Life Technologies) in PBS containing 0.5% Triton X-100. Pre-treated sections were incubated overnight at 4°C with the primary antibodies listed in **Table 1** diluted in PBS containing 0.5% Triton X-100. When indicated, sections were also processed for double-label staining. Double-labeling experiments with anti-cleaved Caspase 3, anti-light chain 3 (LC3), anti-γ-aminobutyric acid transporter-1 (GAT-1), and anti-disabled-1 (Dab1) antibodies, which are all made in rabbit, were performed as previously published (Catalani et al., 2009; Amato et al., 2017). Briefly, sections were first incubated with anti-LC3 antibody for 3 h at room temperature and then in anti-rabbit fluorescein-conjugated Fab fragment antibody (**Table 1**) for 1.5 h at room temperature. Subsequently, the slides were incubated in anti-rabbit unlabeled Fab fragment antibody (**Table 1**) overnight at 4°C and then with anti-cleaved Caspase 3, anti-GAT-1 or anti-Dab1 antibodies. Following washes in PBS, the sections were incubated in the appropriate Alexa Fluor secondary antibodies (Life Technologies) in PBS containing 0.5% Triton X-100 and 5% BSA, for 1.5 h at room temperature. The slides were coverslipped with Fluoroshield Mounting Medium containing DAPI (Abcam, Cambridge, United Kingdom). Incubation in secondary antibody alone was performed as negative control. Images were acquired using a 40× objective by a Zeiss LSM 710 confocal microscope (Carl Zeiss, Oberkochen, Germany). Final images were sized and optimized for contrast and brightness using Adobe Photoshop (Adobe Systems, Mountain View, CA, United States).

### ERG Recordings

Retinal function was examined with scotopic full-field ERG recorded from PD17 anesthetized mice. Before ERG testing, mice were dark adapted for a minimum of 16 h and their manipulation was done under dim red light. Pupils were dilated with 0.5% atropine and a heating pad was used to keep the body temperature at 38°C. The electrophysiological signals were recorded through silver/silver chloride ring electrodes inserted under the lower eyelids. The cornea was intermittently irrigated with saline solution to prevent clouding of the ocular media. Electrodes in each eye were referred to a needle electrode inserted subcutaneously at the level of the corresponding frontal region. The ground electrode was placed on the tail. The electrodes were connected to a two-channel amplifier and ERG responses were evoked by flashes of different light intensities ranging from -3.4 to 1 log cd-s/m^2^ generated through a Ganzfeld stimulator (Biomedica Mangoni, Pisa, Italy). Responses were collected simultaneously from both eyes, amplified at 1,000 gain and filtered with a bandpass of 0.2 to 500 Hz before being digitized at 5 kHz rate with a data acquisition device (Biomedica Mangoni). ERG responses were first analyzed to evaluate the amplitude of a- and b-waves. The amplitude of the a-wave was measured at a fixed time of 8 ms after stimulus onset to minimize contamination from non-photoreceptoral contributions (Robson et al., 2003). The b-wave amplitude was measured from the trough of the a-wave to the peak of the b-wave or, if no a-wave was present, from the prestimulus baseline. Subsequently, ERG responses were analyzed to evaluate the amplitude of oscillatory potentials (OPs). To this aim, ERG responses recorded at stimulus intensities ranging from -1 to 1 log cd-s/m^2^ were digitally filtered with a bandpass of 65–300 Hz to eliminate the a- and b-wave interference and to avoid the 60 Hz line noise. Of the five OPs that can generally be isolated from the mouse ERG, only OP2, OP3, and OP4 were analyzed as OP1 and OP5 could not be accurately measured at PD17 (Vessey et al., 2011). For each OP, the trough-to-peak amplitude was measured and the amplitudes of each wavelet were added to create the sum OPs (SOPs = OP2 + OP3 + OP4; Bresnick and Palta, 1987). Mean amplitudes of ERG responses were plotted as a function of increasing light intensities.

### Statistics

Statistical significance was evaluated using One-way analysis of variance (ANOVA) followed by Newman–Keuls’ multiple comparison post-test or two-way ANOVA followed by Bonferroni’s multiple comparison post-test as appropriate. The results were expressed as means ± SEM of the indicated *n*-values. Prism 5 (GraphPad Software, Inc., La Jolla, CA, United States) was used to analyze data. Differences with *P* < 0.05 were considered statistically significant.

## Results

### Propranolol Affects Apoptosis- and Autophagy-Related Proteins

**FIGURE 1:**
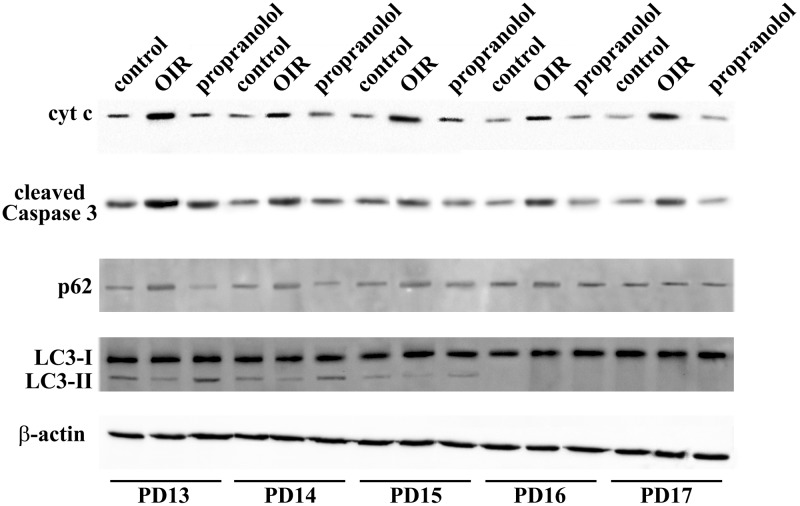
Expression of apoptotic and autophagic markers in the retina. Representative blots for cytochrome c, cleaved Caspase 3, p62 and LC3 as evaluated by western blot analysis using β-actin as the loading control, were shown. Mice were sacrificed every day between PD13 and PD17.

**FIGURE 2:**
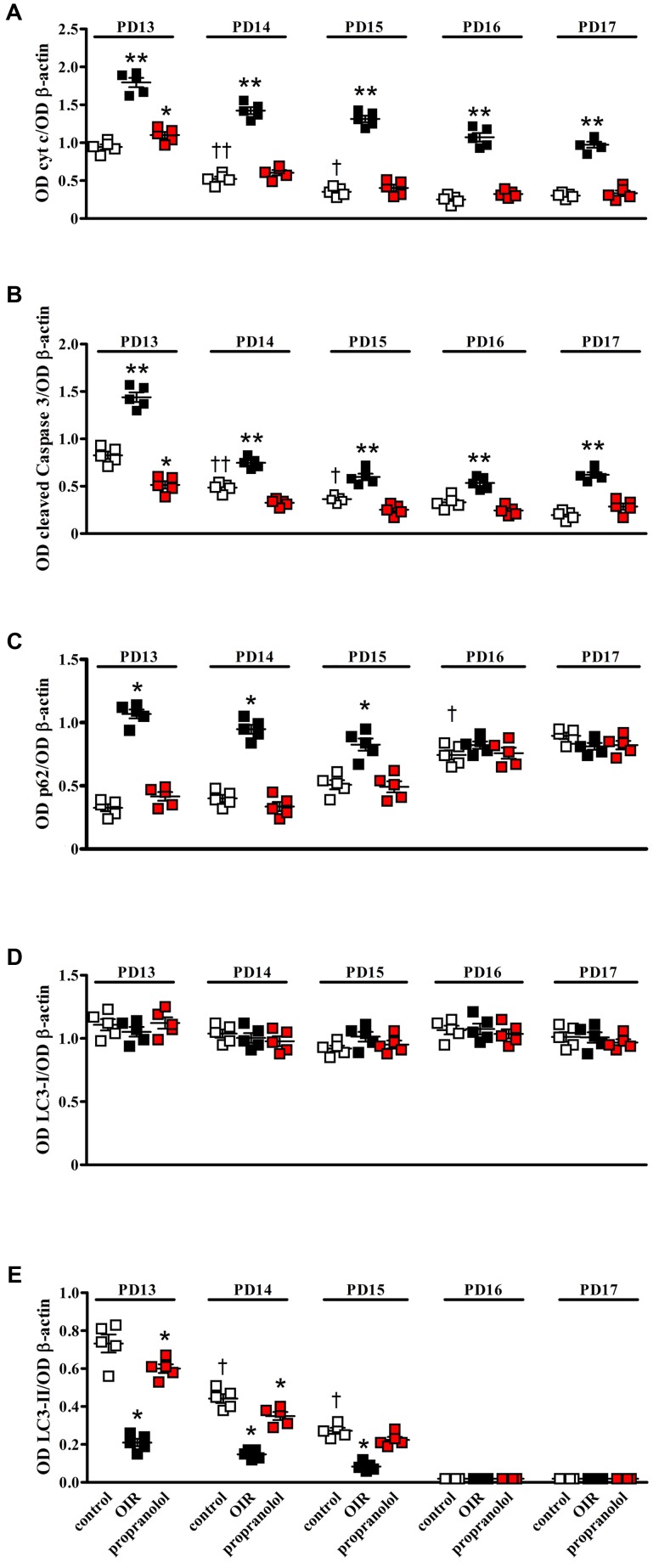
Effects of propranolol on apoptotic and autophagic markers in the retina. Protein levels evaluated by the densitometric analysis of the blots depicted in **Figure 1**. Expression of cytochrome c **(A)** and cleaved Caspase 3 **(B)**. In controls (white), their levels significantly decreased from PD13 to PD15 to then remain constant. In OIR mice (black), apoptotic markers were consistently higher than in controls. Propranolol (red) reduced the levels of apoptotic markers toward recovering their normoxic values. ^∗^*P* < 0.05, ^∗∗^*P* < 0.001 vs. the respective control values; ^†^*P* < 0.05, ^††^*P* < 0.001 vs. control values measured at PD14 or PD13, respectively (two-way ANOVA followed by Bonferroni’s multiple comparison post-test). Expression of p62 **(C)**, LC3-I **(D)**, and LC3-II **(E)**. In controls, the levels of p62 remained low from PD13 to PD15 to then increase at PD16, whereas the levels of LC3-II progressively decreased from PD13 to PD15 to become almost undetectable at PD16. In OIR mice, levels of p62 were higher, while levels of LC3-II were lower, than in controls at PD13, PD14 and PD15. Propranolol reduced p62 while increased LC3-II at PD13, PD14, and PD15 toward recovering their control values. The levels of LC3-I remained constant over time in any experimental condition. ^∗^*P* < 0.001 vs. the respective control values; ^†^*P* < 0.001 vs. control values measured on the previous day (two-way ANOVA followed by Bonferroni’s multiple comparison post-test). Data are presented as scatter plots with mean ± SD.

Western blot analysis was performed using antibodies that bind acknowledged hallmarks of apoptosis, i.e., cytochrome c and the cleaved (active) Caspase 3 or autophagy, i.e., sequestosome 1 (p62) and LC3. Among autophagic markers, the protein p62 is a cargo of ubiquitinated proteins that accumulates in the presence of defective autophagy. LC3 is recruited from the cytosol and associates with the phagophore early in autophagy; during this process LC3 is lipidated and converted from a slow migrating form (LC3-I) in a fast migrating form (LC3-II). Since the lipidation of LC3 may be the result of both induction and suppression of autolysosomal maturation, the measurement of p62 is a useful method to distinguish whether autophagosome accumulation is due to autophagy induction or rather to the inhibition of autophagy steps (Klionsky et al., 2016). Levels of cytosolic cytochrome c, cleaved Caspase 3, p62 and LC3 were evaluated in retinas from PD13 to PD17. Representative blots are shown in **Figure 1**. **Figures 2A,B** shows the densitometric analysis of the blots relative to apoptotic markers in the retina. In controls, their levels significantly decreased from PD13 to PD15, to then remain constant. A similar trend was achieved in retinas of OIR mice although apoptotic markers were consistently higher than in controls. Of interest, propranolol reduced the levels of cytosolic cytochrome c and cleaved Caspase 3 toward recovering their control values. **Figures 2C–E** shows the densitometry analysis of the blots relative to autophagy markers. In control retinas, p62 expression progressively increased from PD13 to PD16/17 while LC3-II decreased. In OIR mice, from PD13 to PD15, retinas were characterized by high levels of p62 and low levels of LC3-II in respect to control retinas, indicating a reduction in the autophagic process. Conversely, propranolol administration decreased p62 and increased LC3-II levels with a trend toward recovering control values. At PD16/17 the expression of p62 was similar in all the experimental groups, while LC3-II levels were almost undetectable. The levels of LC3-I remained constant over time in any experimental condition. Taken together, these results indicate that, in retinas of OIR mice, the propranolol-induced decrease of apoptosis is paralleled by autophagy activation.

### Propranolol Affects the Expression Pattern of Apoptosis and Autophagy Markers

**FIGURE 3:**
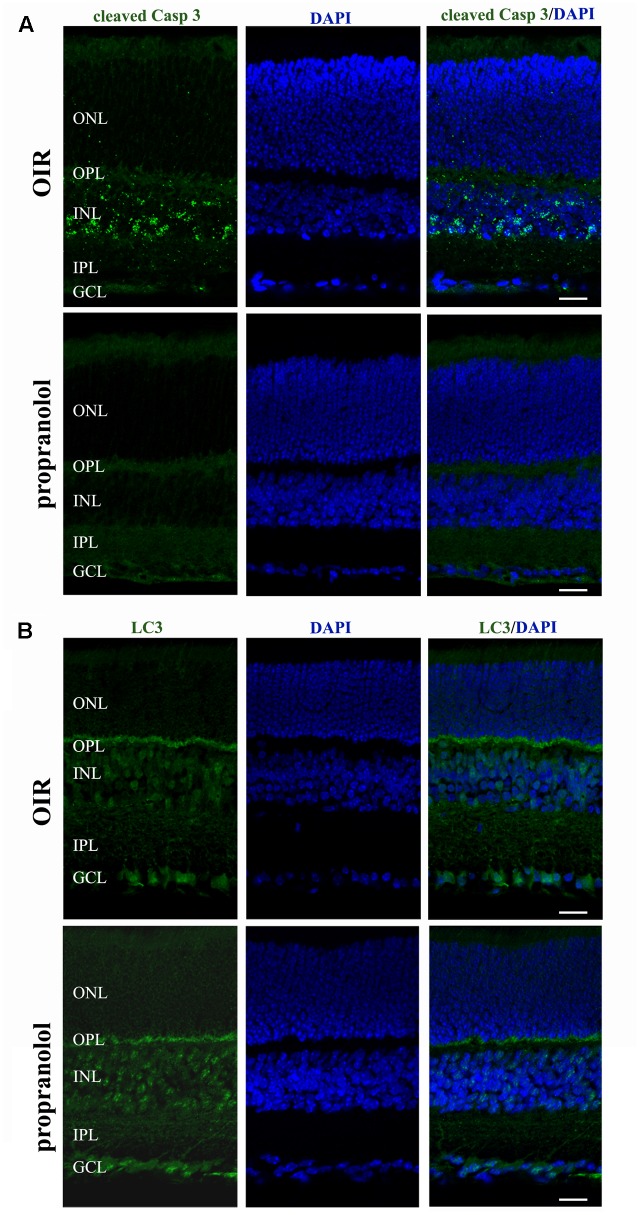
Propranolol effects on the expression pattern of apoptosis and autophagy markers in the retina. Representative confocal images showing the pattern of cleaved Caspase 3 **(A)** and LC3 **(B)** immunofluorescence in retinal sections at PD13, both untreated OIR and propranolol-treated mice. Retinal layers are visualized with DAPI counterstain. Scale bar, 20 μm.

**FIGURE 4:**
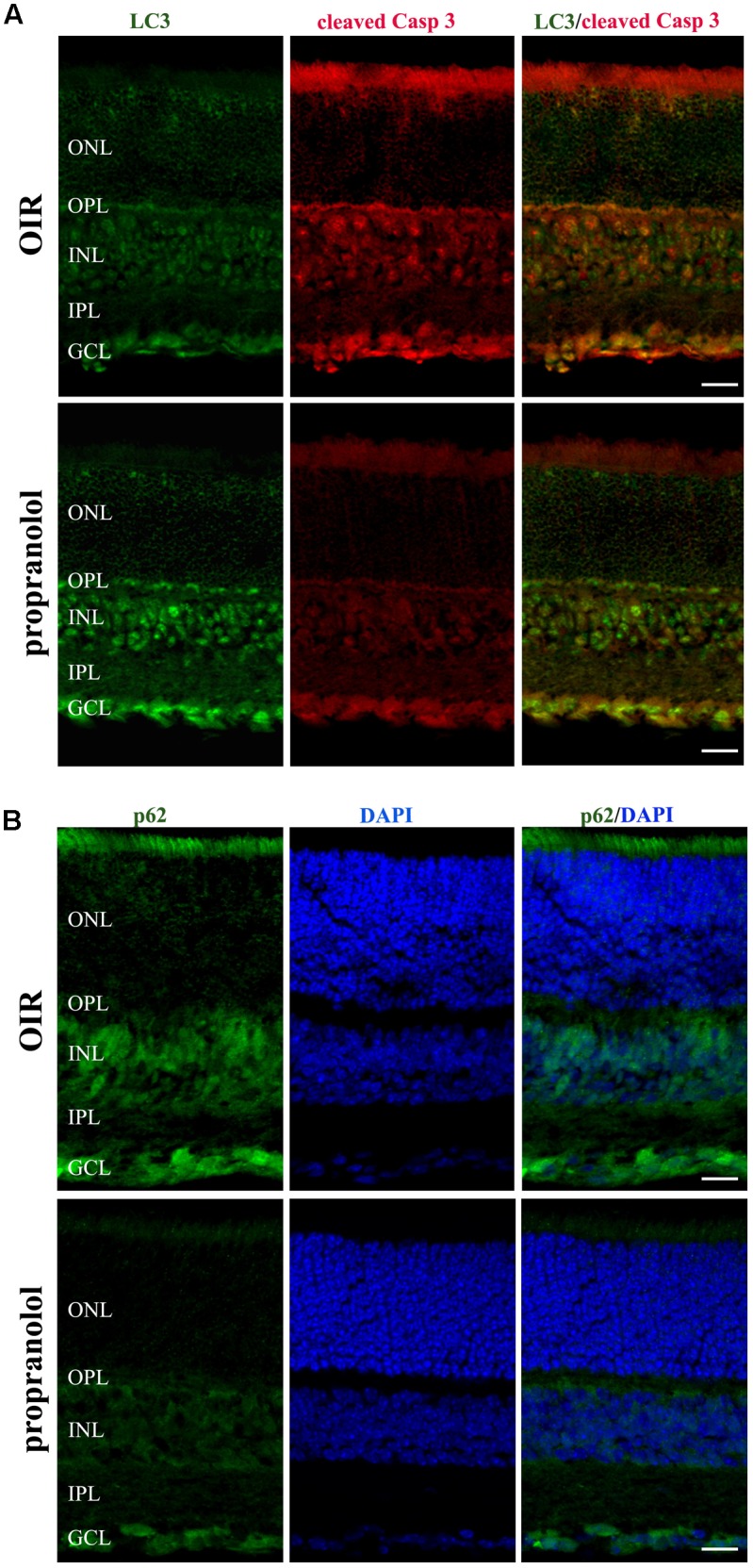
Propranolol effects on the expression pattern of apoptosis and autophagy markers in the retina. **(A)** Double-label immunofluorescence using antibodies directed to LC3 and cleaved Caspase 3. **(B)** Immunostaining showing the pattern of p62. Retinal layers are visualized with DAPI counterstain. The representative confocal images are collected in retinal sections at PD13, both untreated OIR and propranolol-treated mice. Scale bar, 20 μm.

The staining of cleaved Caspase 3, LC3, and p62 was evaluated by immunofluorescence confocal microscopy in retinal sections at PD13. As shown in **Figure 3A**, cleaved Caspase 3 immunolabelling in OIR mice was expressed by cell bodies and fibers mainly localized to the inner nuclear layer (INL), and rare staining for active Caspase 3 was observed in the outer nuclear layer (ONL), the outer plexiform layer (OPL), the inner plexiform layer (IPL) and the ganglion cell layer (GCL). In contrast, the immunolabeling of cleaved Caspase 3 was almost undetectable in retinal sections from propranolol-treated mice. The imaging study of LC3 signals revealed a different pattern between retinal sections of untreated and propranolol-treated OIR mice (**Figure 3B**). In particular, after propranolol administration, LC3 staining clearly changed from diffuse to more intense and punctate, a characteristic of autophagosome buildup when LC3 bound to the autophagosomes. Noteworthy, autophagosomes mainly localized to numerous cell bodies in the INL and GCL; LC3 clustering was also observed in the OPL and IPL. The presence of LC3 immunoreactivity was also investigated in the active Caspase 3-positive cells. As shown in **Figure 4A**, co-localization experiments in untreated OIR mice revealed an high degree of double-immunostained profiles, i.e., diffuse LC3 and cleaved Caspase 3, thus suggesting the close association of cell death and low autophagy; instead, retinal cells of propranolol-treated OIR mice upregulating LC3 expression did not express active Caspase 3. In agreement with western blot data, retinas of OIR mice displayed a strong p62 immunofluorescence, mainly localized to INL and GCL where immunofluorescent profiles were characterized by immunolabeled puncta (**Figure 4B**). Conversely, the presence of immunostaining patterns of p62 clearly decreased in propranolol-treated OIR mice that is consistent with an up-regulation of autophagy.

**FIGURE 5:**
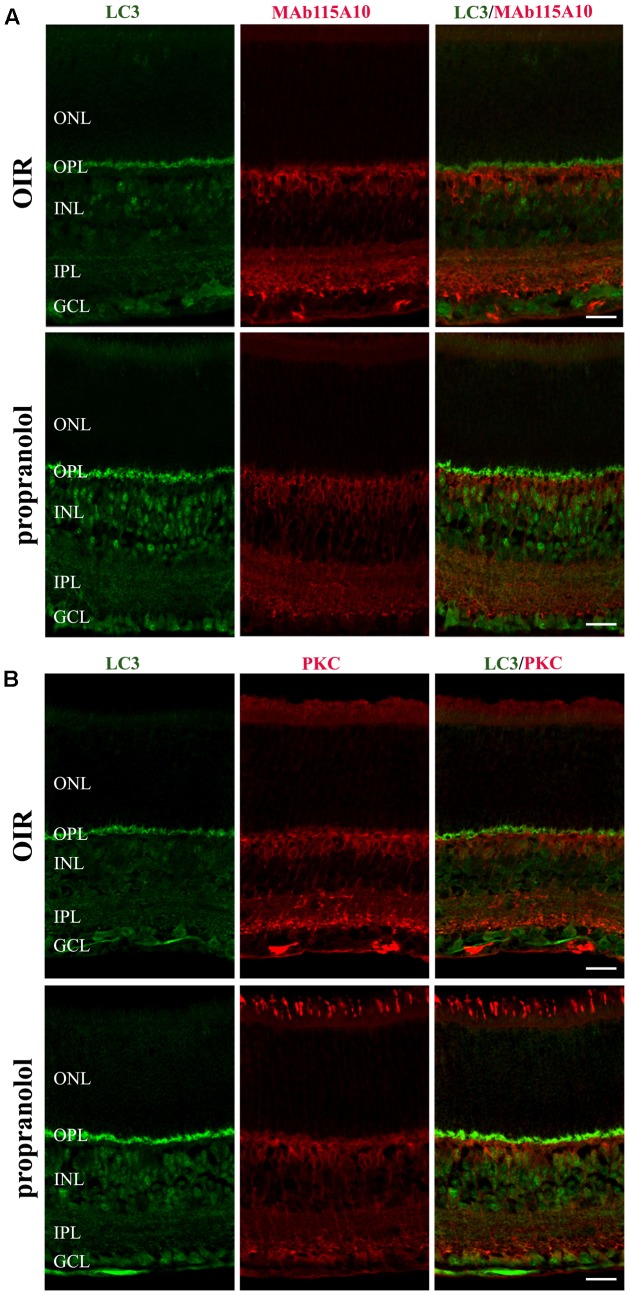
Localization of LC3 staining in the retina. Double-label immunofluorescence using antibodies directed to LC3 and MAb115A10 **(A)** or LC3 and PKC **(B)**. The representative confocal images are collected in retinal sections at PD13, both untreated OIR and propranolol-treated mice. Scale bar, 20 μm.

**FIGURE 6:**
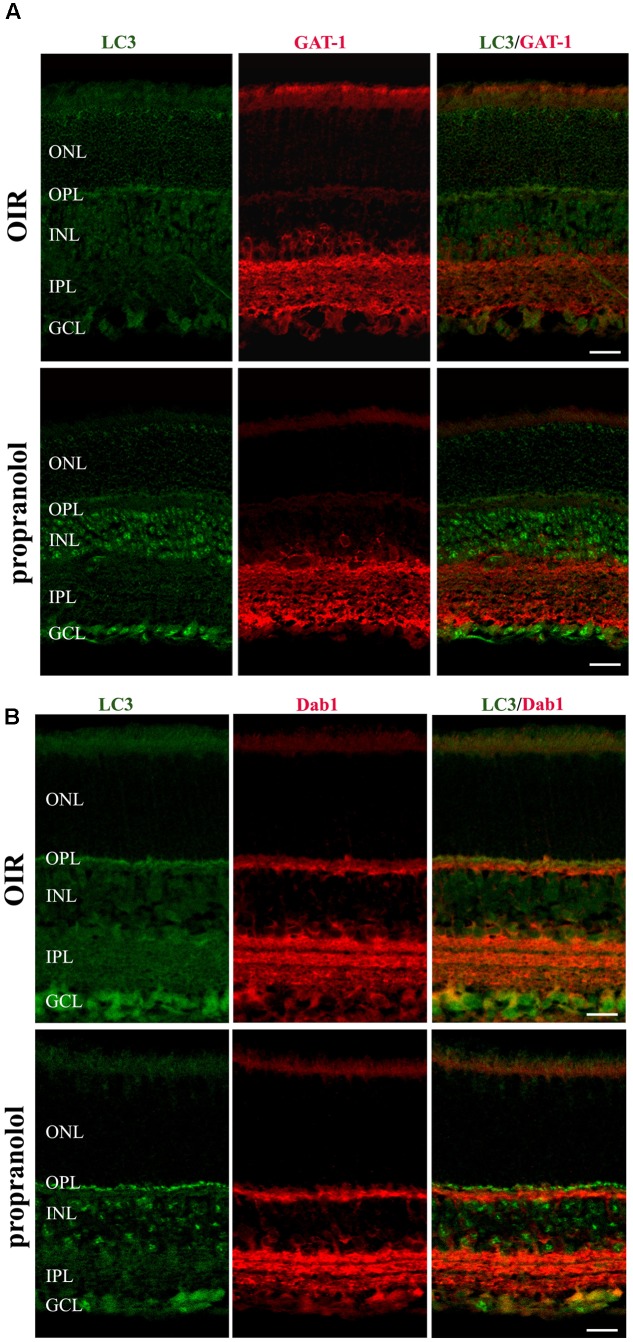
Localization of LC3 staining in the retina. Double-label immunofluorescence using antibodies directed to LC3 and GAT-1 **(A)** or LC3 and Dab1 **(B)**. The representative confocal images are collected in retinal sections at PD13, both untreated OIR and propranolol-treated mice. Scale bar, 20 μm.

**FIGURE 7:**
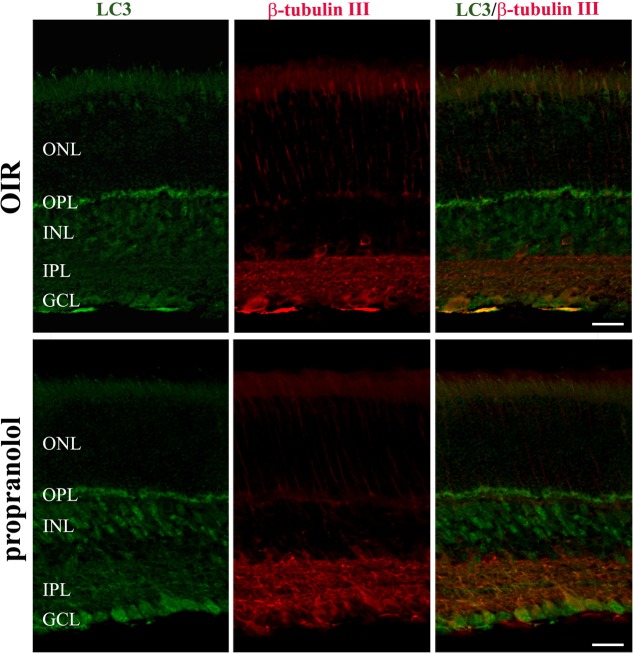
Localization of LC3 staining in the retina. Double-label immunofluorescence using antibodies directed to LC3 and β-tubulin III. The representative confocal images are collected in retinal sections at PD13, both untreated OIR and propranolol-treated mice. Scale bar, 20 μm.

To better characterize the neuroretina cells affected by LC3 clustering in the presence of propranolol at PD13, double-labeling immunofluorescence experiments were then performed using LC3 antibody in conjunction with different markers of retinal cell populations. In particular, MAb115A10 antibody recognizes an antigen expressed by ON-type bipolar cells (which include ON-cone bipolar cells and rod bipolar cells) in the mouse retina while PKC, GAT-1, Dab1 and β-tubulin III antibodies were used to label rod bipolar cells, GABAergic amacrine cells, glycinergic AII amacrine cells and ganglion cells, respectively (Watanabe et al., 1991; Haverkamp and Wassle, 2000; Rice and Curran, 2000; Catalani et al., 2007; Casini, 2008; Cervia et al., 2008, 2012; Amato et al., 2017). Confocal microscopy images revealed that the majority of LC3-punctate in the distal INL were MAb115A10 positive (**Figure 5A**). A certain degree of co-localization was also observed between aggregated LC3 and PKC (**Figure 5B**). In addition, some LC3 clustered cells in the proximal INL co-localizing with GAT-1 and Dab1 staining could be observed (**Figure 6**). Of interest, the staining pattern of aggregated LC3 puncta and β-tubulin III in the GCL was almost superimposable (**Figure 7**).

### Propranolol Modulates Akt-mTOR Autophagy Pathway

**FIGURE 8:**
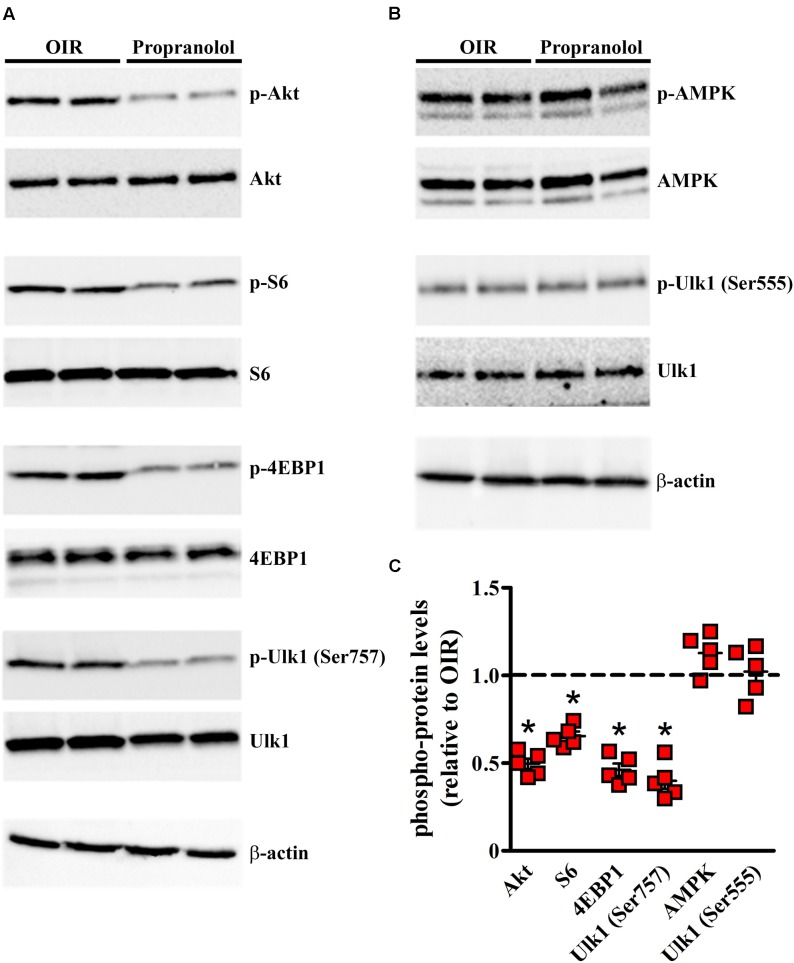
Propranolol effects on autophagy signaling. Activation levels of anti- and pro-autophagic protein kinases were evaluated in the retina by western blot experiments **(A,B)** and their respective densitometric analysis **(C)**. Propranolol reduced the levels of pAkt, pS6, p4EBP1, and pUlk1 at Ser757 while did not affect the levels of pAMPK and pUlk1 at Ser555. The ratio between the expression of phosphorylated protein and the respective total protein is presented as scatter plots with mean ± SD. ^∗^*P* < 0.01 vs. OIR mice values (one-way ANOVA followed by Newman–Keuls’ multiple comparison post-test). β-actin was also used as the loading control.

In another set of experiments, different autophagy signaling molecules were evaluated. Among the critical signaling connections governing autophagy, the mammalian target of rapamycin (mTOR), when is activated by protein kinase B (PKB/Akt), drives (directly and indirectly) the phosphorylation of autophagy controlling proteins including S6, 4EBP1, and Ulk1 at Ser757 site (Klionsky et al., 2016). The activation of this system down-regulates autophagy. On the other hand, the autophagy may be induced through the phosphorylation of Ulk1 at different sites, including Ser555, mediated by the activation of AMPK (Egan et al., 2011; Klionsky et al., 2016). In order to investigate the autophagy pathways mediating propranolol effects, western blot experiments were performed in retinas of OIR mice at PD13, when the effects of relative hypoxia on the autophagy machine are maximal, by assessing the phosphorylation status of either the anti-autophagic molecules Akt, S6, 4EBP1, and Ulk1 at Ser757 site or the pro-autophagic molecules AMPK and Ulk1 at Ser555 site. As shown in the representative blots of **Figures 8A,B** and the densitometric analysis of **Figure 8C**, propranolol significantly reduced the phosphorylation of Akt, S6, 4EBP1, and Ulk1 at Ser757 site without affecting the phosphorylation of AMPK and Ulk1 at Ser555 site.

### Autophagy Pathway Is Responsible of Propranolol-Induced Neuroprotection

**FIGURE 9:**
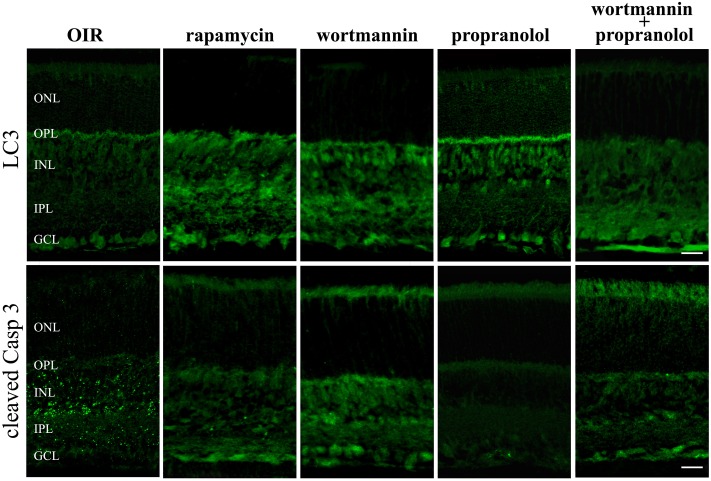
LC3 and active Caspase 3 retinal staining in the presence of autophagy modulators. Immunofluorescence using antibodies directed to cleaved Caspase 3 and LC3 in retinas of OIR mice both in the absence and in the presence of the autophagy stimulator rapamycin or the autophagy inhibitor wortmannin. Propranolol was administered in untreated and wortmannin-treated OIR mice. The representative confocal images are collected in retinal sections at PD13. Scale bar, 20 μm.

To get functional information on the pro-autophagic effects of propranolol, the negative regulator of mTOR rapamycin and the class III PI3 kinase inhibitor wortmannin have been used as positive and negative regulators of autophagy, respectively (Vakifahmetoglu-Norberg et al., 2015). At PD12 OIR mice were intravitreal injected with drugs and the immunofluorescence analysis by confocal microscopy in retinal sections was performed at PD13. As shown in **Figure 9**, rapamycin increased LC3 staining/clustering and inhibited the expression of cleaved Caspase 3, thus mimicking propranolol actions in retinas of OIR mice. In contrast, the administration of wortmannin clearly decreased the effects of propranolol on both LC3 and Caspase 3 immunostaining. In summary, these results argued that the effects of propranolol on retinal apoptosis depend, at least in part, on its activity on the autophagosome system.

### Propranol Effects on ERG

**FIGURE 10:**
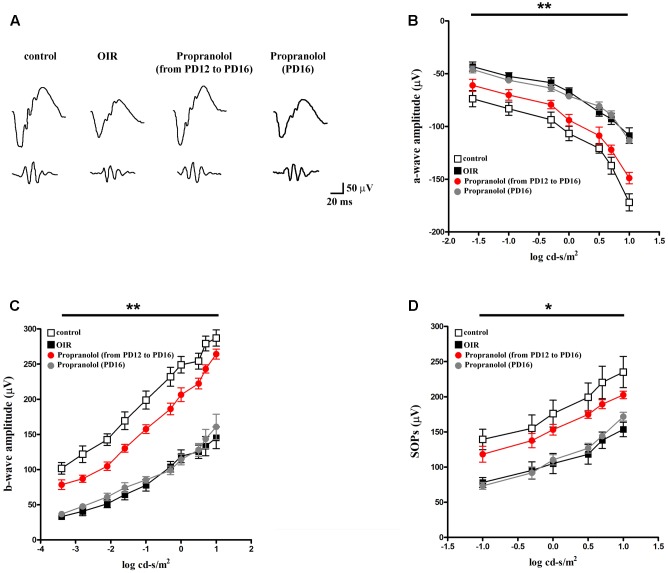
Propranolol effects on retinal functions. **(A)** Representative ERG waveforms in controls, untreated OIR and propranolol-treated mice recorded at light intensities of –1, 0, and 1 log cd-s/m^2^, at PD17. **(B–D)** a-wave, b-wave and SOP amplitudes in controls (white), untreated (black), propranolol-treated from PD12 to PD16 (red), and propranolol-treated at PD16 (gray) OIR mice at increasing light intensities. Repeated propranolol administration, from PD12 to PD16, restored a-, b-wave amplitudes and SOPs to levels that were not significantly different from those in controls. ^∗^*P* < 0.01, ^∗∗^*P* < 0.001 vs. controls (two-way ANOVA followed by Bonferroni’s multiple comparison post-test).

We determined whether the effects of propranolol on apoptosis and autophagy were accompanied by recovered visual dysfunction by recording ERG responses to full-field light flashes. Representative mixed a-, b-waves, and OPs recorded from control and OIR mice either untreated or propranolol-treated are shown in **Figure 10A**. In **Figures 10B,C**, a- and b-wave amplitudes averaged as a function of increasing light intensities are reported. An increase in a- and b-wave amplitudes with increasing stimulus intensity was observed. A clear a-wave developed at a light intensity of approximately -1.6 log cd-s/m^2^. As shown in **Figure 10D**, in control mice SOPs increased as the light intensity was increased. Consistent with previous findings (Martini et al., 2011; Vessey et al., 2011; Dal Monte et al., 2012a, 2015; Lulli et al., 2015), in OIR, vehicle-treated mice displayed significantly reduced a-wave amplitudes (at light intensities ranging from -1.6 to 1 log cd-s/m^2^; **Figure 10B**), b-wave amplitudes (at light intensities ranging from -3.4 to 1 log cd-s/m^2^; **Figure 10C**) and SOPs (at light intensities ranging from -1 to 1 log cd-s/m^2^; **Figure 10D**). Chronic propranolol, i.e., administered from PD12 to PD16, recovered a- and b-wave amplitudes, and SOPs to values that did not significantly differ from those of controls whereas acute propranolol (i.e., injected 1 day before the ERG recordings) did not ameliorate visual dysfunction (**Figures 10B–D**).

## Discussion

Propranolol is one of the first BAR blockers developed. It blocks BAR1 and BAR2, although it seems to be more selective for BAR2 than for BAR1 (Baker, 2010). It also binds to BAR3, but with an affinity which is about two log units lower than that of BAR1 or BAR2 (Baker, 2010; Cernecka et al., 2014). Although off target effects of propranolol have been previously described (Ahrens-Nicklas et al., 2009), results in BAR1/2 knock out mice seem to indicate BAR1 and/or BAR2 as propranolol targets in the retina (Dal Monte et al., 2014). We have previously demonstrated that, in OIR mice, BAR blockade counteracts retinal damage in response to hypoxia thus identifying unexplored modulatory effects by pre-existing drugs (Casini et al., 2014). The present study significantly broadens these aspects characterizing the impact of BAR1/2 blockade in retinal cell death/survival mechanisms. We found that propranolol acts by reducing retinal cell apoptosis while stimulating the autophagy machine. The complex interplay between apoptosis and autophagy would restore retinal cell protection thus contributing to ameliorative effects of propranolol on impaired visual function.

### Propranolol Inhibits Apoptosis and Activates Autophagy

Oxygen induced retinopathy mice model is characterized by apoptotic events that reach a peak in the early phase of ischemia to decline until PD17 (Narayanan et al., 2011; Casini et al., 2014). The present results indeed show that apoptotic markers are higher in retinas of OIR mice than in controls, but progressively decrease from PD13 to PD15 to then remain constant until PD17, in agreement with previous reports indicating that apoptosis decreases by the end of the second postnatal week (Vecino et al., 2004). In this respect, apoptotic DNA fragmentation culminates at PD14 and decreases at PD17 (Sennlaub et al., 2002; Narayanan et al., 2011). The present finding that, in OIR mice, upregulated levels of active Caspase 3 are expressed mainly in the INL, suggests that apoptotic death involves amacrine, bipolar, horizontal and/or Müller cells, whose nuclei are in the INL. This result is in line with the finding that, in OIR mice, TUNEL-positive cells are detected in the INL at PD14 (Sennlaub et al., 2002). In addition, our results show that some neurons in the GCL are positive for active Caspase 3 immunolabelling, indicating that few ganglion cells undergo apoptosis in OIR mice. Of interest, BAR1/2 blockade by propranolol reverses the OIR-induced apoptotic effects thus inhibiting apoptotic death in retinal neurons, suggesting that the retinal norepinephrine overdrive, which has been demonstrated in OIR mice (Dal Monte et al., 2012b), plays a key role in triggering apoptotic death of retinal cell populations. In this respect, an anti-apoptotic activity of propranolol has been demonstrated in staurosporine-treated human neuroblastoma cells (Mikami et al., 2008) or in the heart of aging mice in which BAR blockade reduces the death of myocardial cells (Hu et al., 2008). On the other hand, propranolol seems to exert an anti-cancer activity by increasing apoptosis in human pancreatic cancer cell lines (Zhang et al., 2009), human hemangioma-derived endothelial cells (Ji et al., 2012) and mouse melanoma cells (Dal Monte et al., 2013a).

As also shown by the present results retinas of OIR mice are characterized by reduced autophagy since the decrease of LC3-II is paralleled by an accumulation of p62 at the early stage of relative hypoxia when neuronal cells start to die (Sennlaub et al., 2002; Narayanan et al., 2011). This is in line with previous findings demonstrating that reduced autophagy is an important pathological feature of several ocular diseases (Russo et al., 2013; Chinskey et al., 2014; Frost et al., 2014; Boya et al., 2016; Chai et al., 2016). For instance, there is indication that autophagosomes and autophagy flux are decreased in AMD patients (Mitter et al., 2012) thus indicating that impaired autophagy plays a role in AMD development (Kaarniranta et al., 2013). Disregulation of autophagy is also associated to the progression of neurodegeneration in animal models of DR (Fu et al., 2016; Lopes De Faria et al., 2016; Piano et al., 2016; Rosa et al., 2016; Amato et al., 2017). The present results are consistent with the fact that OIR-associated reduction in autophagy may result in a decreased protective mechanism essential for retinal cell survival in response to injury.

Furthermore, our data indicate an inhibition of autophagy over time, in line with the finding that retinal autophagy processes are time-dependently regulated during postnatal development (Kim et al., 2010). The fact that the early hypoxic phase is characterized by reduced autophagy suggests an early BAR activation in response to the ischemic insult. The additional finding that BAR blockade recovers autophagy to levels comparable to controls is indicative of an efficient action of propranolol counteracting OIR effects. In this respect, propranolol increases autophagy in rat cardiomyocytes (De Meyer and Martinet, 2009) and in human prostate and breast cancer cells (Brohee et al., 2015). Importantly, our results indicate that, in OIR mice, BAR1/2 blockade stimulates autophagy in neuronal cell types committed to die by apoptosis since propranolol increases autophagosome formation mainly in the INL and GCL. Further, LC3/cleaved Caspase 3 co-staining experiments demonstrated that apoptotic cells have low autophagic activity and those cells that enhance their autophagosome formation do not undergo apoptosis. In particular, propranolol displayed pro-autophagic activity on different neuronal cell types, as for instance bipolar cells (including ON-type bipolar cells) and some amacrine (both belonging to GABAergic and glycinergic population) and ganglion cells.

As also shown here, propranolol inhibits the Akt-mTOR pathway in retinas of OIR mice without any modulation of the AMPK signaling. In line with our results, there is evidence that propranolol-induced stimulation of autophagy occur via the inhibition of Akt and S6 phosphorylation (Brohee et al., 2015). In addition, propranolol reduces norepinephrine-induced Akt phosphorylation in human hemangioma-derived endothelial cells (Pan et al., 2015) and norepinephrine-induced S6 phosphorylation in rat pinealocytes (Ho et al., 2003). Noteworthy, the use of specific modulators of autophagy suggests that the propranolol-induced activation of autophagy in OIR mice, likely through the down-regulation of mTOR signaling, plays a key role to inhibit apoptotic cell death of retinal neurons. These protective mechanisms may be explained through the complex interplay between apoptosis and autophagy (Boya et al., 2005; Moscat and Diaz-Meco, 2009; Oral et al., 2016). In this line, in models of optic nerve transection, activation of autophagic processes reduces retinal ganglion cell apoptosis, whereas autophagy inhibition increases ganglion cell susceptibility to apoptosis (Rodriguez-Muela et al., 2012). Accordingly, in the rat retina, ischemia reduces autophagy, while autophagy blockade causes ganglion cell death (Russo et al., 2011). In addition, increased autophagy has been shown to reduce death of rodent photoreceptors and retinal pigment epithelial cells while opposite effects have been achieved by decreased autophagy (Mitter et al., 2014; Rodriguez-Muela et al., 2015; Shelby et al., 2015; Yao et al., 2015). Recently, in an *ex vivo* model of early DR the protective effects of increased autophagy have been demonstrated in populations of bipolar, amacrine and ganglion cells committed to die by apoptosis, thus revealing the antithetic role of apoptosis and autophagy and highlighting their equilibrium from which neuronal survival is likely to depend (Amato et al., 2017).

Overall, our results demonstrate that BAR1/2 are key regulators of retinal apoptosis/autophagy, and that propranolol preserves neuronal cells from apoptotic death. In this respect, triggering autophagosome formation led to propranolol-induced decrease of retinal apoptosis, thus suggesting that an efficient stimulation of autophagy by BAR1/2 blocking may be an effective way to treat neurodegeneration.

### Propranolol Recovers ERG Dysfunction

Interacting with apoptosis and/or autophagy thereby restoring the balance between cell death and cell survival may be a strategy to prevent ERG alterations. For example, in experimental models of retinal damage, inhibiting apoptosis has been shown to prevent visual dysfunction (David et al., 2011; Choudhury et al., 2015; Huang et al., 2015; Wu et al., 2015). In addition, recovering autophagy dysregulation restores ERG in rodent models of retinal diseases (Kunchithapautham et al., 2011; Okamoto et al., 2016). Here, we show that BAR antagonism with chronic (from PD12 to PD16), but not acute (one shot at PD16), propranolol treatment recovers the reduced amplitude of all the ERG components in OIR mice. This demonstrates that, when administered at the beginning of retinal damage when the hypoxia-induced dysregulation of apoptotic/autophagic processes occurs, propranolol is able to efficiently restore retinal function. This is in line with the finding that chronic administration of ICI 118,551, a selective BAR2 blocker, ameliorates retinal function by an associate beneficial effect on both photoreceptors and post-receptor cells in the neuroretina (Martini et al., 2011). On the other hand, in rat models of DR, BAR agonism has been reported to recover ERG amplitude through an anti-apoptotic action (Jiang et al., 2010). In addition, although at high doses, BAR blockade causes dysfunctional ERG in healthy rats and rabbits (Jiang and Steinle, 2010; Nourinia et al., 2015). Taken together, these findings suggest that the role of BAR-acting drugs may be species-specific and may also depend on the administration route, dosage, and/or involve different downstream pathways in relation to the specific physio-pathological states.

In OIR mice, the reduction of the a-wave may be an index of a decreased input to bipolar cells, which are known to generate the b-wave together with Müller cells thus, subsequently, determining reduced signal transduction from bipolar to amacrine cells, which are likely to participate in the generation of OPs (Wachtmeister, 1998). Of interest, OPs are generated by cells of the INL and are sensitive indicators of visual dysfunction (Fulton et al., 2009). In this respect, the present ERG findings are consistent with the anti-apoptotic/pro-autophagic role of BAR1/2 blockade thus indicating that propranolol may prevent OIR-induced retinal cell loss mostly affecting the signal transduction of INL cells.

## Conclusion

**FIGURE 11:**
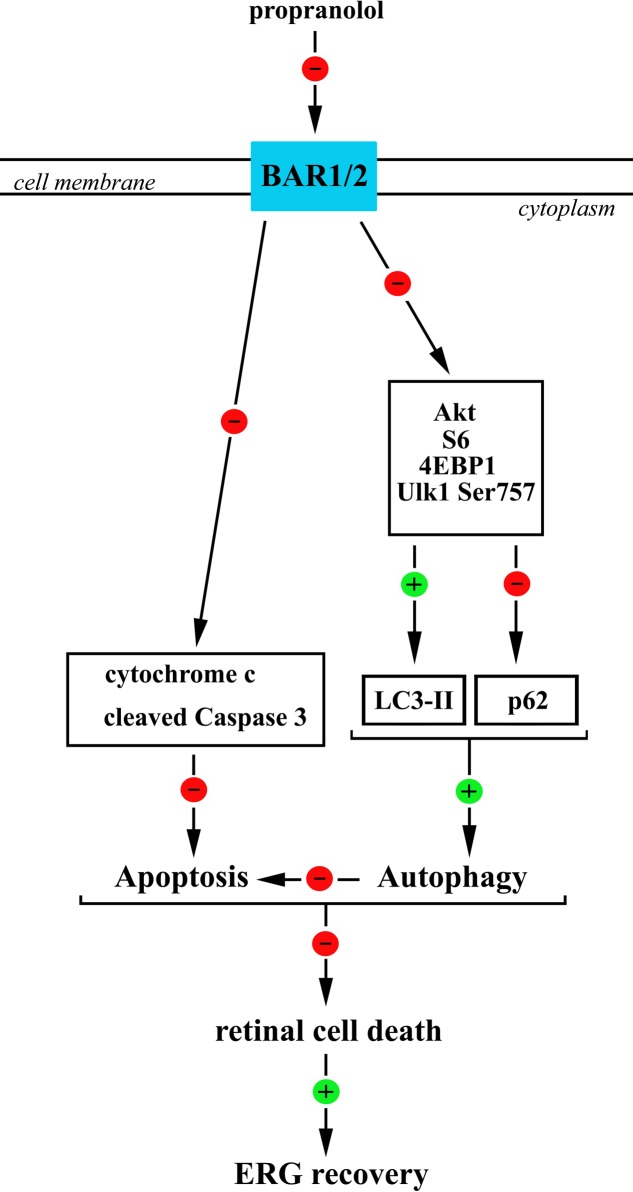
A schematic diagram representing the proposed mechanisms by which propranolol reduces apoptosis/autophagy neuronal ratio in the retina therefore recovering ERG dysfunction. Propranolol, by blocking BARs, causes a reduced phosphorylation of anti-autophagic molecules, including Akt, S6, 4EBP1, and Ulk1 at Ser 757 site that would result in reduced levels of p62 and increased levels/clustering of LC3-II both indicative of an increased autophagosome formation. BAR blockade would also cause a reduction in the levels of the apoptotic molecules cytochrome c and cleaved (active) Caspase 3 thus counteracting OIR-associated apoptotic processes. We hypothesize that the stimulated autophagy triggers anti-apoptotic events leading to ameliorative effects of propranolol on the damaged retina. In this scenario, the coordinated increase in autophagy and decrease in apoptosis may play a key role to reduce retinal cell death and ameliorate visual performance.

If one considers the complex crosstalk between apoptosis and autophagy, then a coordinated regulation of these processes should be the key determinant for retina protection from degenerative events therefore resulting in recovered retinal function. Our data indicate that the increased autophagy in retinal neurons after BAR1/2 blockade leads to neuroprotection, i.e., decreased apoptosis/autophagy cell ratio, and recovered visual dysfunction (**Figure 11**).

## Author Contributions

MC was the responsible of animal handling and sample collection, performed ERG testing and data processing, initiated the project, supervised all experiments on a daily basis and contributed to elaborating the text. FL contributed to animal handling, ERG testing, data processing and sample collection. EC performed immunofluorescence experiments and data processing. LF contributed to the experimental design and to work discussion. MDM performed western blot experiments, analyzed the data, contributed to article writing and work supervision. DC performed western blot analysis, designed, coordinated and supervised the experiments, analyzed the data and contributed to writing the article. PB coordinated the whole experimental and analysis work and contributed to article writing.

## Conflict of Interest Statement

The authors declare that the research was conducted in the absence of any commercial or financial relationships that could be construed as a potential conflict of interest.
