# Supplementary material for: The Beta Adrenergic Receptor Blocker Propranolol Counteracts Retinal Dysfunction in a Mouse Model of Oxygen Induced Retinopathy: Restoring the Balance between Apoptosis and Autophagy
Source: Front Cell Neurosci. 2017 Dec 12;11:395. doi: 10.3389/fncel.2017.00395 (PMC5770647; doi:10.3389/fncel.2017.00395)
Supplement: Supplementary file 2 [file Image_2.PDF]

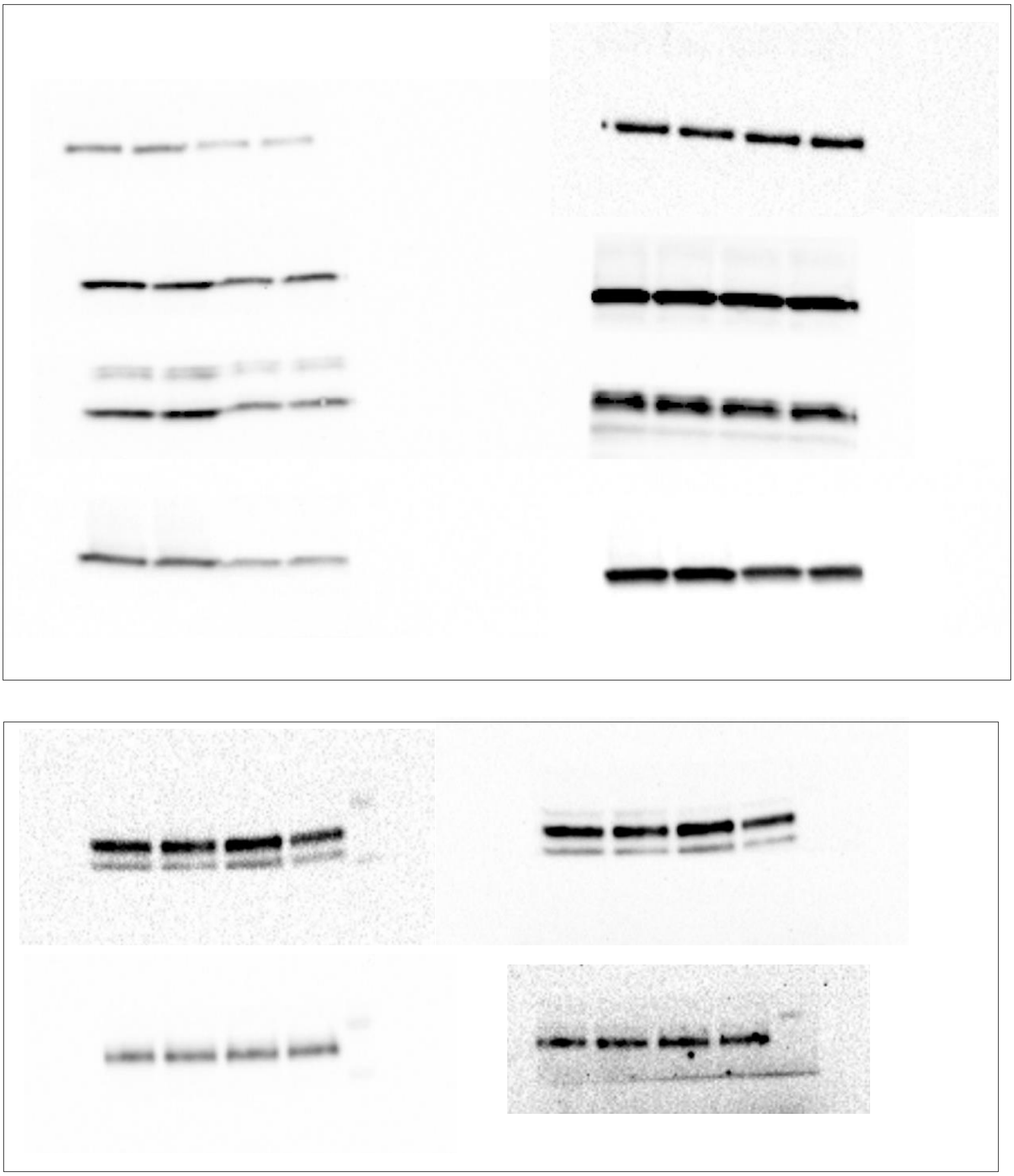

Supplementary Figure 2. Original images of representative western blot from Figure 8. Please note that the original full blots have been cut in small pieces before the incubation with the appropriate antibody. The pictures represent the unmodified digitized images as originally acquired.
